# Associations of Physical Behaviours and Behavioural Reallocations with Markers of Metabolic Health: A Compositional Data Analysis

**DOI:** 10.3390/ijerph15102280

**Published:** 2018-10-17

**Authors:** Gregory J. H. Biddle, Charlotte L. Edwardson, Joseph Henson, Melanie J. Davies, Kamlesh Khunti, Alex V. Rowlands, Thomas Yates

**Affiliations:** 1 Diabetes Research Centre, University of Leicester, Leicester LE5 4PW, UK; ce95@le.ac.uk (C.L.E.); jjh18@le.ac.uk (J.H.); melanie.davies@uhl-tr.nhs.uk (M.J.D.); kk22@le.ac.uk (K.K.); alex.rowlands@leicester.ac.uk (A.V.R.); ty20@le.ac.uk (T.Y.); 2 NIHR Leicester Biomedical Research Centre, Leicester LE5 4PW, UK; 3 Health Sciences, University of Leicester, Leicester LE1 7RH, UK; 4 Leicester Diabetes Centre, University Hospitals of Leicester, Leicester LE5 4PW, UK; 5 NIHR Collaborations for Leadership in Applied Health Research and Care (CLAHRC) East Midlands, Leicester LE5 4PW, UK

**Keywords:** sleep, sedentary behaviour, physical activity, time use, metabolic health

## Abstract

Standard statistical modelling has shown that the reallocation of sitting time to either standing or stepping may be beneficial for metabolic health. However, this overlooks the inherent dependency of time spent in all behaviours. The aim is to examine the associations between physical behaviours and markers of metabolic health (fasting glucose, fasting insulin, 2-h glucose, 2-h insulin, Homeostasis Model Assessment of Insulin Sensitivity (HOMA-IS), Matsuda Insulin Sensitivity Index (Matsuda-ISI) while quantifying the associations of reallocating time from one physical behaviour to another using compositional analysis. Objectively measured physical behaviour data were analysed (*n* = 435) using compositional analysis and compositional isotemporal substitutions to estimate the association of reallocating time from one behaviour to another in a population at high risk of type 2 diabetes mellitus (T2DM). Stepping time was associated with all markers of metabolic health relative to all other behaviours. Reallocating 30 min from sleep, sitting, or standing to stepping was associated with 5–6 fold lower 2-h glucose, 15–17 fold lower 2-h insulin, and higher insulin sensitivity (10–11 fold via HOMA-IS, 12–15 fold via Matsuda-ISI). Associations of reallocating time from any behaviour to stepping were maintained for 2-h glucose, 2-h insulin, and Matsuda-ISI after further adjusting for body mass index (BMI). Relocating time from stepping into sleep, sitting, or standing was associated with lower insulin sensitivity. Stepping time may be the most important behavioural composition when promoting improved metabolic health in adults at risk of T2DM.

## 1. Introduction

A 24-h day constitutes a finite period of time made up of a series of physical behaviours. These behaviours, typically defined as sleep, sedentary behaviour, light-intensity physical activity (LIPA), and moderate-to-vigorous-intensity physical activity (MVPA), have previously been described as having independent associations with health. For example, short sleep duration has been associated with increased risk of metabolic syndrome [1], whereas both short and long sleep duration has been associated with increased risk of mortality [2]. Sedentary behaviour, defined as sitting, reclining, or lying behaviour with low energy expenditure (≤1.5 metabolic equivalents) during waking hours [3,4], has been associated with increased risk of type 2 diabetes mellitus (T2DM), cardiovascular disease, and mortality [5,6,7,8,9]. Physical activity has been shown to reduce morbidity and mortality [10,11,12]. Research has examined reallocating one behaviour to another, showing positive results for increasing movement (stepping, LIPA, and MVPA) by decreasing sedentary time [13,14,15,16,17,18,19]. With the growing interest in sedentary behaviour, there has been an increase in the use of thigh-worn accelerometers that can measure the postures of sitting and standing, rather than infer sitting time through lack of movement, which has been done with waist-worn accelerometers [20]. The activPAL is increasingly being used in sedentary behaviour and physical activity research [21]. A small number of isotemporal substitution studies have utilised the activPAL accelerometer for physical behaviour measurement to date [14,15,16]. All three employed a traditional isotemporal substitution approach, developed by Mekary et al. [22], to model the associations of reallocating time from sitting to standing or stepping. The evidence shows that reallocating time from sitting to standing or stepping is associated with favourable health markers [14,15,16]. However, there is a growing body of evidence using a different analytical approach, this being compositional data analysis.

Traditional linear regression and isotemporal substitution analyses treat time-use data as absolute values, whereas compositional analyses treat time-use data as relative values. The development of a compositional data analysis approach has allowed for an alternative analytical approach, which addresses the intrinsic codependency of physical behaviours to account for the time spent in each behaviour by treating each behaviour as a composite of a finite whole [23,24]. Instead of treating data as continuous and defining them in standard real space, compositional data are defined in a constrained space, or simplex. Two studies have previously compared these two analytical approaches [23,25]. Both studies noted a broad agreement between the analytical approaches, with both outlining the importance of time spent in MVPA. However, both report how a compositional approach produced asymmetric results for the reallocation of behaviours. This means that when time was reallocated to MVPA from sedentary behaviour, the estimated associations were less than the inverse reallocation, this being from MVPA to sedentary behaviour. To date, no studies, to the authors’ knowledge, have utilised a compositional data analysis approach in a population at high risk of developing T2DM with robust measures of physical behaviours and biochemical markers of metabolic health. It is therefore necessary to examine whether compositional data analysis approaches can be used to provide additional insight into the associations of substituting one physical behaviour for another with markers of metabolic health in an “at risk” population.

The aims of this study were to examine the associations between time spent in four physical behaviours (sleep, sitting, standing, and stepping) and markers of glucose regulation and insulin sensitivity, and to quantify the association of reallocated time from one physical behaviour to another with markers of glucose regulation and insulin sensitivity.

## 2. Materials and Methods

### 2.1. Participants

The sample consisted of participants taking part in the Walking Away from Diabetes cluster randomized control trial, the methods and results of which have been reported previously [26,27]. In brief, 10 general practitioner (GP) practices were recruited from the East Midlands, United Kingdom (UK), within which participants were identified on general practice databases using an automated version of the Leicester Risk Assessment Tool [28], designed to calculated an individual’s risk of developing T2DM. Adults aged 30–75 years scoring above the 90th centile of the risk score were invited to take part. Data collected at 36-month follow up (2013–2014) were utilised for this analysis because this is the first time point participants wore the activPAL accelerometer. There was no difference between the intervention and the control group for the primary outcome (i.e., objectively measured physical activity via the ActiGraph GT3X) at 36-month follow up [26]; therefore, data were analysed using the combined cohort.

### 2.2. Sleep, Sitting, Standing, and Stepping Measurement

Movement behaviour was measured using the activPAL3 device (PAL Technologies, Glasgow, UK), an accelerometer attached to the thigh. This device determines time spent sitting or lying and time upright. Upright time is then classified as standing or stepping. The device was initialized using the manufacturer’s software under default settings. Waterproofing of the device was achieved using a nitrile sleeve and Hypafix dressing. The activPAL3 has been validated and shown to be highly accurate for use in adult populations [20,29,30,31,32]. Participants were requested to wear the device for seven consecutive days, 24 h a day, on the midline anterior aspect of the thigh, attached using a piece of waterproof Hypafix dressing. activPAL data were processed using a validated automated algorithm in STATA (StataCorp, LP, Texas, TX, USA), described in detail elsewhere [33,34]. This algorithm is designed to isolate waking wear time from “sleep” (time in bed), prolonged non-wear and invalid data, with no distinction made. As such, sleep time is inferred by subtracting waking wear time from 24 h. As this may include non-wear and invalid data, heat-maps of data were examined to identify any non-wear and invalid data that may have occurred on valid days. To be included in this analysis, a minimum of four valid wear days were required and days with at least 10 h of waking wear data, >500 steps, and <95% spent in any one behaviour were included. Self-reported sleep was also measured by asking participants their average sleep duration over the past seven days. This was used in a sensitivity analysis (see Section 2.6).

### 2.3. Demographic, Anthropometric, and Medication Status

Body mass (Tanita TBE 611, Tanita, West Drayton, UK) and height were measured to the nearest 0.1 kg and 0.5 cm, respectively. Information on current smoking status, medication, and ethnicity was obtained following an interview administered protocol with a health care professional.

### 2.4. Biomarker Measures

Glucose and insulin were measured via venous blood samples collected fasted and 2 h post-challenge. Glucose samples were analysed using a glucose oxidase method on the Beckman Auto Analyzer (Beckman, High Wycombe, UK) within a laboratory at the Leicester Royal Infirmary. Plasma samples were frozen at −80 °C for insulin analysis at the end of data collection using an enzyme immunoassay (80-INSHU-E01.1, E10.1 Alpco Diagnostics 26G Keewaydin Drive, Salem, NH, USA) at a specialist laboratory by Unilever R&D, Bedfordshire, UK. For this analysis, two-hour glucose and two-hour insulin were used. The homeostasis model assessment of insulin sensitivity (HOMA-IS) and the Matsuda insulin sensitivity index (Matsuda-ISI) were both calculated to estimate insulin sensitivity [35,36], as follows:

HOMA–IS = 1/HOMA-IR = 22.5/(G_0_ · I_0_),

Matsuda-ISI = 1000/squt(G_0_ · I_0_ · G_120_ · I_120_).

### 2.5. Data Inclusion

Of the total sample attending 36-month follow-up (*n* = 530), a total of 435 (82%) had activPAL data and were included in this analysis. Ethical approval for the use of the activPAL was not granted at the start of the 36-month visits, which resulted in some missing data. Details of these data (with and without activPAL) were reported previously [14]. Briefly, those providing invalid data were more likely to be male and have higher fasting insulin.

### 2.6. Statistical Analysis

All statistical analyses were performed using the R statistical system, version 3.4.3. All analyses followed published guidelines for conducting compositional data analysis and isotemporal substitution [23,24]. Descriptive statistics were calculated (means, standard deviations, medians, interquartile ranges (IQR), and percentages) to describe the population. Descriptive statistics were also stratified by tertiles of stepping time. Physical behaviour was outlined as geometric means, normalized to represent minutes of each behaviour in a 1440 min day. A variation matrix was then calculated to represent the codependence of each physical behaviour with each other [23]. Values closer to zero represent higher codependency, whereas values closer to one represent lower codependency.

Linear regression models using compositional methods were conducted to examine the associations of time spent sleeping, sitting, standing, and stepping on glucose, insulin, and markers of insulin sensitivity. Isometric log-ratios (ILRs), rather than individual movement behaviours, were used as predictor variables. This methodological approach allowed all four physical behaviours to act as the independent variable, while taking into account the relative time spent in the other physical behaviours. In addition, the overall model fit was provided (R^2^). Model 1 was adjusted for basic demographic and lifestyle factors (age, sex, ethnicity, smoking status, β-blocker use, statin use, and family history of diabetes), whereas Model 2 was further adjusted for body mass index (BMI). In addition to generating associations for each variable within the composition, the authors of this study also used the compositional nature of the analysis to model the association of substituting 30 min of one behaviour for another using methods detailed previously [23,24]. The physical behaviours were first normalised to 24 h a day, then the predicted percentage difference in each outcome resulting from reallocating time was calculated, with 95% confidence intervals (CI) at an alpha of equal to or less than 0.05. Furthermore, three sensitivity analyses were conducted: first (Models 2a and 2b), to assess whether the results were affected if self-reported sleep time was used instead of algorithm-derived sleep time; second, to assess whether associations with markers of insulin sensitivity (HOMA-IS and Matsuda-ISI) differed between long and short sleepers using a median split; and third, to assess whether the associations of 30 min reallocations differed when conducted using a traditional isotemporal substitution approach. Self-reported sleep time was used instead of algorithm-derived sleep time for Model 2a, whereas Model 2b used the difference between self-reported sleep time and algorithm-derived sleep time and manually added it to the sitting time Model 2b. Model 3a analysed the short sleepers subset, whereas Model 3b analysed the long sleeper subset. Model 4 (adjusted for age, sex, ethnicity, smoking status, β-blocker use, statin use, and family history of diabetes) and Model 5 (additionally adjusted for BMI) utilised the traditional isotemporal substitution approach.

### 2.7. Ethics Statement

All participants gave their informed consent for inclusion before they participated in the study. The study was conducted in accordance with the Declaration of Helsinki, and the protocol was approved by the National Health Service (NHS) National Research Ethics Service–Nottingham Research Ethics Committee 2 (09/H0408/32).

## 3. Results

Table 1 displays the characteristics of those included in the analysis; the average age and BMI were 66 years of age (±7.4) and 31.4 kg/m^2^ (±5.3), respectively. Mean HbA1c was 5.7% ± 0.5, with 15.4% of participants with an HbA1c within the prediabetes range of 6 to 6.49%.

Table 2 shows the geometric means of the physical behaviours that make up the daily time-use composition. The largest proportion of the day was spent sitting (39.6%), closely followed by sleep (35.6%). Stepping time only took up 6.7% of time, whereas participants stood for 18.1% of time.

Table 3 shows the variation matrix between each physical behaviour. Providing confidence intervals for geometric means is not meaningful as the variance of a single composite does not consider the codependency between composites. Therefore, to highlight the relative dispersion structure of the composites, a variation matrix is calculated. The highest level of codependency was observed between sleep and sitting time, whereas the lowest levels of codependency were observed between all behaviours and stepping and between sitting and standing.

### 3.1. Linear Regression Models

The results of the linear regression are presented in Table 4. All assumptions of linear regression were met. In Model 1, the proportion of time spent stepping was associated with fasting glucose (0.95, *p* = 0.023), fasting insulin (0.72, *p* = 0.003) two-hour glucose (0.82, *p* ≤ 0.0001), two-hour insulin (0.53, *p* = 0.009), HOMA-IS (1.49, *p* = 0.001), and Matsuda-ISI (1.70, *p* = 0.005). The proportion of time spent sleeping was only associated with a lower Matsuda-ISI value (0.66, *p* = 0.033). The proportion of time spent sitting and standing was not associated with any marker. The associations for stepping time and two-hour glucose (0.83, *p* ≤ 0.0001), two-hour insulin (0.57, *p* = 0.002), and Matsuda-ISI (1.51, *p* = 0.004) persisted after further adjustment for BMI, as did the association for time spent sleeping and Matsuda-ISI (0.66, *p* = 0.033). However, the associations between the proportion of time stepping and fasting glucose (0.96, *p* = 0.062), fasting insulin (0.84, *p* = 0.114) and (HOMA-IS (1.26, *p* = 0.057) were attenuated. Results were similar in Models 2a and 2b of the sensitivity analyses using the different definition of sleep (see Table S1). Results of Models 3a and 3b showed a similar pattern for the associations with stepping time, whereas the pattern of associations differed for sleep and standing time between short and long sleepers. However, no associations observed in Models 3a and 3b were statistically significant, with the exception of stepping time and Matsuda-ISI for long sleepers (see Table S2).

### 3.2. Isotemporal Substitutions

**Figure 1:**
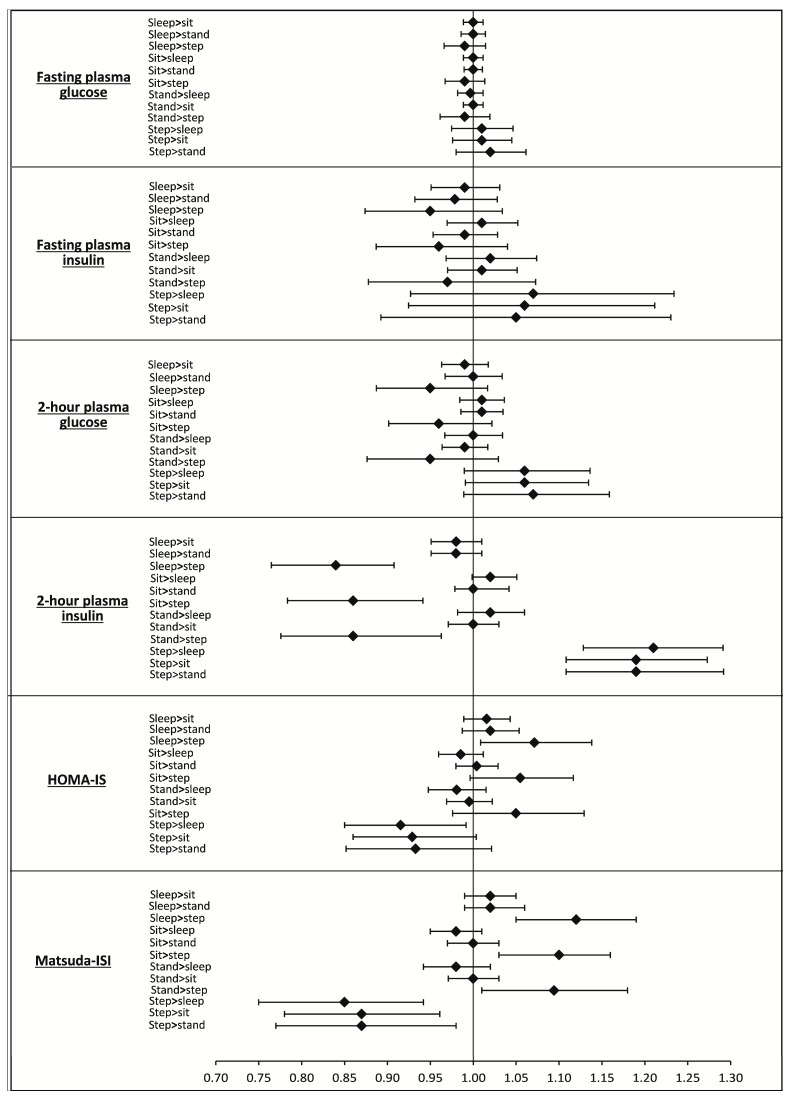
Fold difference (95% CI) per 30 min/day reallocation. Adjusted for age, sex, ethnicity, smoking status, β-blocker use, statin use, family history of diabetes, and BMI (Model 2 only).

The results of the isotemporal substitutions are presented in Table 5 and Figure 1 (Model 2 only). After adjustment for demographics, smoking history, family history of T2DM, and medication status (Model 1), reallocating 30 min from sleep, sitting, or standing to stepping resulted in favourable associations for 2-h glucose (−5 fold [sleep], −4 fold [sitting], −5 fold [standing]), 2-h insulin (−17 fold, −15 fold, −15 fold), HOMA-IS (13 fold, 13 fold, 12 fold), and Matsuda-ISI (12 fold, 10 fold, 9 fold). Equally, reallocating 30 min from stepping time to either sleep, sitting, or standing had detrimental associations with two-hour glucose (6 fold, 6 fold, 7 fold), two-hour insulin (21 fold, 19 fold, 19 fold), HOMA-IS (11 fold, 10 fold, 10 fold), and Matsuda-ISI (−15 fold, −13 fold, −13 fold). After additionally adjusting for BMI (Model 2), results persisted for all reallocations with the exception of sitting or standing to stepping and stepping to sitting or standing for HOMA-IS. These results were broadly similar to the results of the traditional isotemporal substitution models (see Table S3). However, only the compositional isotemporal substitutions showed asymmetrical associations for the reallocation of stepping time to sleep, sitting, or standing when compared with the associations for the reallocation of sleep, sitting, or standing to stepping.

These reallocations are asymmetrical, meaning the reallocation of 30 min from one behaviour to another does not necessarily result in the same association as the inverse reallocation. For example, the reallocation of 30 min from sitting to stepping was associated with 5 fold lower two-hour glucose, whereas the inverse reallocation, 30 min from stepping to sitting, was associated with a 6 fold higher value. Reallocating time from stepping to all other physical behaviours had a larger detrimental association with metabolic markers than the inverse reallocations. This asymmetrical relationship was observed for every significant reallocation. Figure 1 shows the asymmetric relationship estimated for these reallocations of physical behaviours. These reallocations should be interpreted as fold differences, meaning any associations must be interpreted in relation to the original value (i.e., the mean of the dependent variable).

## 4. Discussion

This study is the first to the authors’ knowledge to utilise a compositional data analysis approach to model the association between physical behaviour, measured via the activPAL, and markers of metabolic health in a population with a high risk of T2DM recruited from primary care, as well as to examine associations of reallocating time in different physical behaviours. Results demonstrated that time spent stepping was associated with markers of metabolic health when accounting for the intrinsic codependency of all physical behaviours, and that reallocating time spent in any other physical behaviour to stepping was associated with favourable associations for two-hour glucose, two-hour insulin, and Matsuda-ISI. Furthermore, reallocating stepping time to sleep, sitting, or standing was associated with larger detrimental associations on two-hour glucose, two-hour insulin, and Matsuda-ISI. These results show the importance of not only increasing time spent stepping or moving, but also maintaining any current stepping or movement time. Time spent sleeping, sitting, and standing were not associated with two-hour glucose, two-hour insulin, or Matsuda-ISI, although sleep time was associated with HOMA-IS (sitting and standing time were not associated). Sitting time was the dominant behavioural composition accounting for 39.6% of the 24-h day.

These results are broadly similar to previous evidence from compositional data analyses [23,25,37,38,39,40], as well as previous isotemporal substitutional analyses using traditional methodologies [13,14,15,16,17]. As with previous evidence, movement appears to be the most influential physical behaviour. The main area of contrast with previous isotemporal analyses was the relationship with standing time, which has previously been shown to be favourably associated with markers of cardiometabolic health [14,15,16,17]. The compositional approach utilised in this study does not support the reallocation of sitting time to standing. As such, the results and subsequent interpretation may differ depending on the analytical approach. There remains mixed evidence, both experimentally and epidemiologically, of the role standing has on metabolic health, and health more broadly [15,41,42,43,44,45,46,47,48,49]. As such, further research is needed to clarify the effect of standing, particularly the effect of reducing sitting time and replacing it with standing in a free-living environment, appropriately accounting for time in all other physical behaviours, over a longer period of time. Further investigations of the association of sleep time with markers of metabolic health are also warranted, as the pattern of associations seemed to differ between short and long sleepers, suggesting sleep time may be associated with increased insulin sensitivity in short sleepers and decreased insulin sensitivity in long sleepers. However, there were no statistically significant associations, therefore further research is required to clarify these issues.

In contrast to the evidence on the benefits of standing, the benefits of stepping are more consistent. Not only have previous isotemporal substitution analyses shown favourable results from the reallocation of sedentary time to movement (stepping, light-intensity physical activity and moderate-to-vigorous-intensity physical activity) [13,14,15,16,17], but all compositional data analyses have demonstrated the importance of movement in relation to all other physical behaviours [23,25,37,38,39,50]. Equally, numerous behaviour change studies have shown benefits from increases in ambulation [51]. For example, a recent meta-analysis summarizing the effect of 38 walking interventions on cardiovascular disease risk factors [51] found positive effects for adiposity, blood pressure, and fasting glucose [51]. The results of the current study further emphasise the importance of stepping time on metabolic health, particularly in high risk populations.

The current findings support previous compositional isotemporal substitution analyses [23,25,37,39,50] suggesting an asymmetric relationship with the reallocation of stepping time. The estimated detrimental results of a reduction in stepping time are greater than the estimated favourable results for the inverse reallocation, that being an increase in stepping time. These asymmetric associations are only observed when conducted via compositional isotemporal substitution. Traditional isotemporal substitution, as reported in Supplementary Table S3, show a symmetrical association for time reallocations. Importantly, these results do not imply that one methodological approach is better than the other, rather the convergence and divergence between methods are highlighted, enabling results of previous and future evidence to be better interpreted. Chastin et al. hypothesises this relationship may be due to two separate factors [23]: first, that time spent in movement is much shorter than time spent sitting, therefore any reallocation of these behaviours constitutes substantially difference percentages of time for each behaviour respectively; second, it is highlighted that deconditioning or weight gain requires lower stimuli (i.e., occurs more easily) than the equivalent conditioning or weight loss [52,53,54]. However, it is also possible that this asymmetric relationship is an artifact of the analysis, as 30 min of sitting will affect the compositions to a lesser extent than 30 min of stepping. When energy expenditure is considered, theoretically, a 30 min reallocation to or from stepping time will have the same size of effect on energy expenditure. Therefore, this asymmetric association needs further investigation.

This study has many strengths; first, the authors utilised a compositional data analysis approach which adjusted appropriately for the codependency of physical behaviours across a finite 24-h day. They also utilised objectively measured physical behaviour via the activPAL3, the first time to their knowledge that this device had been used in this form of analysis. Robust measures of two-hour glucose and insulin were also used which allowed them to accurately examine insulin sensitivity. The sample is also both a strength and a limitation, because the high risk nature of the study’s cohort represents the population that is most likely to receive and benefit from future interventions based around the results of studies such as this, whereas results cannot necessarily be generalized to the general population. There are a few limitations in this study, primarily the inability to measure sleep directly within the activPAL data. However, results also remained largely unchanged when utilising self-reported sleep time. The cross-sectional nature of the data limits the authors’ ability to infer causality. The cross-sectional nature of the cohort also means that the study estimates the associations with metabolic health makers if there was a population shift in physical behaviour rather than individual reallocations (change) of behaviour [23].

## 5. Conclusions

This study emphasises the importance of increasing and maintaining stepping time at the expense of any other physical behaviour. Considering that the largest proportion of time during the day is spent sitting, this demonstrates a key area of focus for future work. These results have important implications for national diabetes prevention programs and for individuals at high risk of developing T2DM. These results also contribute to the growing evidence utilising a compositional data analysis approach which allows for the association of all physical behaviours to be assessed while adjusting for their intrinsic codependency. Ultimately, this approach provides a more detailed understanding of physical behaviours and their relationship with health. Further compositional analysis is encouraged to grow the body of evidence surrounding physical behaviours, with a particular focus on longitudinal and interventional studies that will enable an assessment of causality.

## Figures and Tables

**Table 1:** Descriptive statistics.

Characteristics	*n* = 435	Stepping Time Tertiles		
		Low	Median	High
Age (years)	66.9 (7.4)	67.3 (8.1)	66.8 (7.0)	66.5 (7.1)
Male (%)	61.7	44.8	65.5	74.5
White European (%)	89.2	89.7	89.0	89.0
Body Mass Index (BMI) (kg/m^2^)	31.4 (5.3)	33.3 (5.6)	31.2 (5.3)	29.9 (4.4)
Using β-Blockers (%)	20.2	14.5	19.3	15.2
Using Lipid-Lowering Medication (%)	32.3	24.8	33.1	35.2
Family History of Diabetes (%)	32.7	31.7	34.5	39.3
Current Smokers (%)	7.3	7.6	5.5	6.2
HbA1c (unit %)	5.7 (0.5)	5.7 (0.5)	5.6 (0.6)	5.7 (0.4)
Normal Range [≤6.0] (%)	79.7	72.2	82.8	84.1
Prediabetes Range [6–6.5] (%)	15.4	19.4	14.5	12.4
Diabetes Range [≥6.5] (%)	4.8	8.3	2.8	3.4
Fasting Plasma Glucose (mmol/L)	5.2 (4.8–5.5)	5.3 (4.9–5.7)	5.0 (4.7–5.4)	5.2 (4.9–5.5)
2-h Plasma Glucose (mmol/L)	5.6 (4.7–6.9)	6.0 (4.9–7.3)	5.4 (4.7–6.3)	5.5 (4.4–6.3)
Fasting Insulin (mU/L)	9.0 (6.3–13.1)	11.0 (7.3–15.1)	8.5 (6.4–12.6)	7.8 (5.4–11.8)
2-h Insulin (mU/L)	44.0 (23.7–79.2)	56.8 (35.9–98.0)	40.8 (22.2–70.2)	36.4 (18.3–65.8)

Continuous variables are presented as mean ± SD, unless non-parametric which are presented as median (IQR). Categorical variables are reported as percentages.

**Table 2:** Geometric means of physical behaviour composites.

Characteristics	Minutes/Day	Percentage of 24 h
Sleep	512.7	35.6
Sitting	570.0	39.6
Standing	261.2	18.1
Stepping	96.1	6.7

Physical behaviour has been normalised to 1440 min.

**Table 3:** Variation matrix.

	Sleep	Sitting	Standing	Stepping
Sleep		0.074	0.159	0.201
Sitting	0.074		0.236	0.255
Standing	0.159	0.236		0.181
Stepping	0.201	0.255	0.181	

Data present the variance in the log ratio between factors. Numbers closer to zero represent factors that have a high level of codependency.

**Table 4:** Compositional linear models showing the association between time spent in different physical behaviours and metabolic biomarkers.

	Sleep	Sitting	Standing	Stepping	Model Fit (R^2^)
**Model 1**					
Fasting glucose	1.01	1.01	1.03	**0.95 ^†^**	0.002
(95% CI)	(0.95; 1.07)	(0.97; 1.05)	(0.99; 1.07)	**(0.91; 0.99)**	
Fasting insulin	1.28	1.14	0.96	0.72 *	0.061
(95% CI)	(0.94; 1.76)	(0.90; 1.44)	(0.82; 1.32)	**(0.58; 0.89)**	
Two-hour glucose	1.11	1.02	1.08	0.82 ***	0.062
(95% CI)	(0.96; 1.27)	(0.92; 1.13)	(0.98; 1.19)	**(0.74; 0.90)**	
Two-hour insulin	1.63	1.11	1.05	0.53 **	0.059
(95% CI)	(0.98; 2.69)	(0.75; 0.61)	(0.74; 1.50)	**(0.37; 0.76)**	
HOMA-IS	0.75	0.89	1.01	1.49 *	0.061
(95% CI)	(0.54; 1.04)	(0.69; 1.14)	(0.70; 1.17)	**(1.18; 1.89)**	
Matsuda-ISI	**0.66**	0.89	1.01	1.70 **	0.087
(95% CI)	**(0.44; 0.97) ^†^**	(0.66; 1.19)	(0.77; 1.33)	**(1.23; 2.23)**	
**Model 2**					
Fasting glucose	1.01	1.01	1.03	0.96	0.009
(95% CI)	(0.95; 1.07)	(0.97; 1.05)	(0.99; 1.07)	(0.92; 1.00)	
Fasting insulin	1.28	1.00	0.92	0.84	0.149
(95% CI)	(0.96; 1.72)	(0.79; 1.26)	(0.74; 1.15)	(0.68; 1.05)	
Two-hour glucose	1.12	1.00	1.08	0.83 **	0.062
(95% CI)	(0.97; 1.28)	(0.83; 1.23)	(0.98; 1.19)	**(0.75; 0.91)**	
Two-hour insulin	1.63	1.05	1.04	0.57 *	0.064
(95% CI)	(0.98; 2.72)	(0.71; 1.56)	(0.73; 1.48)	**(0.40; 0.80)**	
HOMA-IS	0.75	1.01	1.05	1.26	0.146
(95% CI)	(0.54; 1.04)	(0.78; 1.30)	(0.83; 1.33)	(1.00; 1.59)	
Matsuda-ISI	**0.66 ^†^**	0.98	1.02	1.51 *	0.133
(95% CI)	**(0.46; 0.96)**	(0.73; 1.32)	(0.78; 1.34)	**(1.15; 1.98)**	

Values for each physical behaviour represent the association for time spent in each movement behaviour relative to all other behaviours (95% confidence interval (CI)). Model 1 adjusted for age, sex, ethnicity, smoking status, beta-blocker use, statin use, and family history of type 2 diabetes. Model 2 additionally adjusted for BMI. Significance levels: *** *p* < 0.0001, ** *p* < 0.001, * *p* < 0.01, ^†^ *p* < 0.05. Significant results are also highlighted in bold.

**Table 5:** Compositional isotemporal substitutions.

	Sleep	Sitting	Standing	Stepping
**Model 1**				
**Fasting glucose**				
*Sleep*		1.00 (1.00; 1.00)	1.00 (1.00; 1.01)	**0.99 (0.98; 1.00)**
*Sitting*	1.00 (1.00; 1.00)		1.00 (1.00; 1.01)	**0.99 (0.98; 1.00)**
*Standing*	1.00 (1.00; 1.00)	1.00 (0.99; 1.00)		**0.99 (0.97; 1.00)**
*Stepping*	**1.02 (1.00; 1.03)**	**1.02 (1.00; 1.03)**	**1.02 (1.00; 1.03)**	
**Fasting insulin**				
*Sleep*		0.99 (0.97; 1.02)	0.98 (0.95; 1.02)	**0.91 (0.86; 0.97)**
*Sitting*	1.01 (0.98; 1.03)		0.99 (0.97; 1.01)	**0.92 (0.87; 0.97)**
*Standing*	1.02 (0.98; 1.05)	1.01 (0.98; 1.04)		**0.93 (0.87; 0.99)**
*Stepping*	**1.13 (1.04; 1.22)**	**1.12 (1.04; 1.21)**	**1.11 (1.02; 1.21)**	
**Two-hour glucose**				
*Sleep*		1.00 (0.98; 1.01)	1.00 (0.99; 1.02)	0.95 (0.92; 0.97)
*Sitting*	1.00 (0.99; 1.02)		1.01 (1.00; 1.02)	0.95 (0.93; 0.98)
*Standing*	1.00 (0.98; 1.01)	0.99 (0.98; 1.00)		0.94 (0.91; 0.98)
*Stepping*	**1.07 (1.04; 1.10)**	**1.06 (1.03; 1.10)**	**1.07 (1.03; 1.11)**	
**Two-hour insulin**				
*Sleep*		0.98 (0.94; 1.02)	0.98 (0.93; 1.03)	**0.83 (0.73; 0.92)**
*Sitting*	1.02 (0.98; 1.06)		1.00 (0.96; 1.04)	**0.85 (0.76; 0.93)**
*Standing*	1.02 (0.97; 1.07)	1.00 (0.96; 1.04)		**0.85 (0.74; 0.96)**
*Stepping*	**0.77 (0.65; 0.89)**	**0.79 (0.68; 0.91)**	**0.79 (0.65; 0.93)**	
**HOMA-IS**				
*Sleep*		1.01 (0.98; 1.04)	1.02 (0.98; 1.05)	**1.11 (1.05; 1.19)**
*Sitting*	0.99 (0.96; 1.02)		1.01 (0.98; 1.03)	**1.10 (1.04; 1.17)**
*Standing*	0.98 (0.95; 1.02)	0.99 (0.97; 1.02)		**1.10 (1.02; 1.18)**
*Stepping*	**0.87 (0.80; 0.94)**	**0.87 (0.81; 0.95)**	**0.88 (0.80; 0.97)**	
**Matsuda-ISI**				
*Sleep*		1.02 (0.98; 1.05)	1.02 (0.98; 1.06)	**1.15 (1.07; 1.22)**
*Sitting*	0.99 (0.95; 1.02)		1.01 (0.98; 1.04)	**1.13 (1.06; 1.20)**
*Standing*	0.98 (0.94; 1.02)	0.99 (0.96; 1.03)		**1.12 (1.04; 1.21)**
*Stepping*	**0.81 (0.71; 0.90)**	**0.82 (0.73; 0.91)**	**0.83 (0.72; 0.94)**	
**Model 2**				
**Fasting glucose**				
*Sleep*		1.00 (0.99; 1.00)	1.00 (1.00; 1.01)	0.99 (0.98; 1.00)
*Sitting*	1.00 (1.00; 1.01)		1.00 (1.00; 1.01)	0.99 (0.98; 1.00)
*Standing*	1.00 (1.00; 1.00)	1.00 (0.99; 1.00)		0.99 (0.98; 1.00)
*Stepping*	1.01 (1.00; 1.03)	1.01 (1.00; 1.03)	1.02 (1.00; 1.03)	
**Fasting insulin**				
*Sleep*		0.99 (0.96; 1.01)	0.98 (0.95; 1.01)	0.95 (0.90; 1.00)
*Sitting*	1.01 (0.99; 1.04)		0.99 (0.97; 1.02)	0.96 (0.91; 1.01)
*Standing*	1.02 (0.99; 1.05)	1.01 (0.98; 1.03)		0.97 (0.90; 1.03)
*Stepping*	1.07 (1.00; 1.15)	1.06 (0.99; 1.14)	1.05 (0.97; 1.14)	
**Two-hour glucose**				
*Sleep*		0.99 (0.98; 1.01)	1.00 (0.99; 1.02)	**0.95 (0.93; 0.98)**
*Sitting*	1.01 (0.99; 1.02)		1.01 (1.00; 1.02)	**0.96 (0.93; 0.98)**
*Standing*	1.00 (0.98; 1.01)	0.99 (0.98; 1.00)		**0.95 (0.92; 0.98)**
*Stepping*	**1.06 (1.03; 1.10)**	**1.06 (1.03; 1.09)**	**1.07 (1.03; 1.11)**	
**Two-hour insulin**				
*Sleep*		0.98 (0.94; 1.02)	0.98 (0.93; 1.03)	**0.84 (0.75; 0.93)**
*Sitting*	1.02 (0.98; 1.06)		1.00 (0.96; 1.04)	**0.86 (0.78; 0.95)**
*Standing*	1.02 (0.97; 1.07)	1.00 (0.96; 1.04)		**0.86 (0.75; 0.97)**
*Stepping*	**1.21 (1.09; 1.33)**	**1.19 (1.07; 1.31)**	**1.19 (1.05; 1.33)**	
**HOMA-IS**				
*Sleep*		1.02 (0.99; 1.04)	1.02 (0.99; 1.05)	**1.07 (1.01; 1.14)**
*Sitting*	0.99 (0.96; 1.01)		1.00 (0.98; 1.02)	1.05 (0.99; 1.12)
*Standing*	0.98 (0.95; 1.01)	1.00 (0.97; 1.02)		1.05 (0.98; 1.13)
*Stepping*	**0.92 (0.85; 0.99)**	0.93 (0.86; 1.00)	0.93 (0.85; 1.02)	
**Matsuda-ISI**				
*Sleep*		1.02 (0.99; 1.05)	1.02 (0.99; 1.06)	**1.12 (1.05; 1.19)**
*Sitting*	0.98 (0.95; 1.01)		1.00 (0.97; 1.03)	**1.10 (1.03; 1.16)**
*Standing*	0.98 (0.94; 1.02)	1.00 (0.97; 1.03)		**1.09 (1.01; 1.18)**
*Stepping*	**0.85 (0.75; 0.94)**	**0.87 (0.78; 0.96)**	**0.87 (0.77; 0.98)**	

Time is reallocated from the behaviours in the rows to the behaviours in the columns. Values represent the factor by which the outcome measure is multiplied by (95 fold confidence interval) for a 30 min difference in the substituted physical behaviour. Bold values indicate a statistically significant estimate to an alpha of 0.05. Model 1 is adjusted for age, sex, ethnicity, smoking status, beta-blocker use, statin use, and family history of type 2 diabetes. Model 2 is additionally adjusted for BMI.

## Supplementary Materials

The following are available online at http://www.mdpi.com/1660-4601/15/10/2280/s1, Table S1: Compositional Linear Models Showing the Association between Time Spent in Different Physical behaviours And Metabolic Biomarkers: Alternative definitions of sleep, Table S2: Compositional Linear Models Showing the Association between Time Spent in Different Physical behaviours And Markers of Insulin Sensitivity: Short Sleepers vs. Long Sleepers, Table S3: Traditional Isotemporal Substitutions.
