# Supplementary material for: Associations of Physical Behaviours and Behavioural Reallocations with Markers of Metabolic Health: A Compositional Data Analysis
Source: Int J Environ Res Public Health. 2018 Oct 17;15(10):2280. doi: 10.3390/ijerph15102280 (PMC6210541; doi:10.3390/ijerph15102280)
Supplement: Supplementary file 1 [file ijerph-15-02280-s001.pdf]

Table S1. Compositional Linear Models Showing the Association between Time Spent in Different Physical behaviours And Metabolic Biomarkers: Alternative definitions of sleep.

	Sleep	Sitting	Standing	Stepping	Model fit (R^2)
**Model 2a**					
Fasting glucose	1.02	0.99	**1.03**	**0.96**	0.01
	(0.96; 1.08)	(0.93; 1.05)	**(0.99; 1.07)**	**(0.92; 1.00)**	
Fasting insulin	1.02	1.15	**0.98**	**0.87**	0.14
	(0.96; 1.08)	(0.91; 1.45)	**(0.79; 1.22)**	**(0.70; 1.08)**	
Two-hour glucose	0.99	1.07	**1.13†**	**0.84****	0.06
	(0.86; 1.14)	(0.95; 1.21)	**(1.02; 1.24)**	**(0.76; 0.93)**	
Two-hour insulin	1.12	1.31	1.14	**0.61***	0.10
	(0.67; 1.86)	(0.87; 1.98)	(0.79; 1.65)	**(0.43; 0.86)**	
HOMA-IS	1.03	1.36	1.11	0.58	0.14
	(0.75; 1.41)	(1.06; 1.76)	(0.87; 1.40)	(0.65; 1.05)	
Matsuda-ISI	1.00	0.77	0.93	**1.40†**	0.12
	(0.68; 1.48)	(0.57; 1.03)	(0.71; 1.23)	**(1.07; 1.85)**	
**Model 2b**					
Fasting glucose	1.01	1.00	1.03	1.03	0.01
	(0.97; 1.52)	(0.96; 1.04)	(0.99; 1.07)	(0.99; 1.07)	
Fasting insulin	1.03	**1.16**	**0.97**	**0.85**	0.15
	(0.81; 1.30)	**(0.97; 1.39)**	**(0.78; 1.20)**	**(0.69; 1.06)**	
Two-hour glucose	0.99	**1.11†**	**1.12†**	**0.83****	0.07
	(0.88; 1.11)	**(1.02; 1.19)**	**(1.01; 1.23)**	**(0.75; 0.91)**	
Two-hour insulin	1.14	1.36	1.11	**0.58***	0.11
	(0.75; 1.72)	(1.00; 1.87)	(0.76; 1.60)	**(0.41; 0.82)**	
HOMA-IS	1.04	1.17	1.00	0.81	0.14
	(0.81; 1.34)	(0.96; 1.43)	(0.79; 1.26)	(0.62; 1.00)	
Matsuda-ISI	0.98	**0.74†**	0.95	**1.46***	0.13
	(0.72; 1.34)	**(0.59; 0.94)**	(0.72; 1.25)	**(1.11; 1.92)**	

Results for each physical behaviour represent the association for time spent in each movement behavior relative to all other behaviours. Adjusted for age, sex, ethnicity, smoking status, β-blocker use, statin use, family history of diabetes and BMI. Significance levels: p<0.0001 = ***, p<0.001 = **, p<0.01 = *, p<0.05 = †

Table S2. Compositional Linear Models Showing the Association between Time Spent in Different Physical behaviours And Markers of Insulin Sensitivity: Short Sleepers vs Long Sleepers.

	Sleep	Sitting	Standing	Stepping	Model fit (R^2)
**Model 3a**					
**Short Sleepers**					
HOMA-IS	1.31	0.85	0.84	1.01	0.01
(95% CI)	(0.63; 2.71)	(0.52; 1.39)	(0.58; 1.21)	(0.72; 1.41)	
Matsuda-ISI	1.21	0.82	0.77	1.31	0.06
(95% CI)	(0.52; 2.81)	(0.46; 1.45)	(0.50; 1.19)	(0.87; 1.98)	
**Model 3b**					
**Long Sleepers**					
HOMA-IS	0.81	0.82	1.14	1.31	0.21
(95% CI)	(0.38; 1.71)	(0.49; 1.36)	(0.78; 1.65)	(0.94; 1.83)	
Matsuda-ISI	0.85	0.74	1.04	**1.52†**	0.20
(95% CI)	(0.37; 1.98)	(0.42; 1.31)	(0.68; 1.60)	**(1.05; 2.21)**	

Results for each physical behaviour represent the association for time spent in each movement behavior relative to all other behaviours. Adjusted for age, sex, ethnicity, smoking status, β-blocker use, statin use, family history of diabetes and BMI. Significance levels: $p<0.0001$ = ***, $p<0.001$ = **, $p<0.01$ = *, $p<0.05$ = †

Table S3. Traditional Isotemporal Substitutions.

	Standing to Sitting	*p* value	Stepping to sitting	*p* value	Stepping to standing	*p* value
**Model 4**						
HOMA-IS	0.99	0.294	0.91	0.002	0.92	0.030
(95% CI)	(0.96; 1.01)		(0.85; 0.97)		(0.85; 0.99)	
Matsuda-ISI	0.99	0.481	0.86	<0.001	0.87	0.002
(95% CI)	(0.96; 1.02)		(0.80; 0.93)		(0.80; 0.95)	
**Model 5**						
HOMA-IS	0.99	0.546	0.95	0.078	0.96	0.206
(95% CI)	(0.97; 1.02)		(0.89; 1.01)		0.89; 1.03)	
Matsuda-ISI	0.99	0.786	0.89	0.002	0.90	0.012
(95% CI)	(0.98; 1.04)		(0.83; 0.96)		(0.82; 0.98)	
	Sitting to standing	*P* value	Sitting to stepping	*P* value	Standing to stepping	*P* value
**Model 4**						
HOMA-IS	1.01	0.294	1.10	0.002	1.09	0.030
(95% CI)	(0.99; 1.04)		(1.04; 1.17)		(1.01; 1.17)	
Matsuda-ISI	1.01	0.481	1.16	<0.001	1.15	0.002
(95% CI)	(0.98; 1.04)		(1.08; 1.24)		(1.05.; 1.25)	
**Model 5**						
HOMA-IS	1.01	0.546	1.06	0.078	1.05	0.206
(95% CI)	(0.98; 1.03)		(0.99; 1.12)		(0.97; 1.13)	
Matsuda-ISI	1.00	0.786	1.12	0.002	1.12	0.012
(95% CI)	(0.98; 1.03)		(1.04; 1.20)		(1.02; 1.21)	

Model 1 adjusted for age, sex, ethnicity, smoking status, beta-blocker use, statin use and family history of type 2 diabetes. Model 2 additionally adjusted BMI.
